# Supplementary material for: Combined Simplified Molecular Classification of Gastric Adenocarcinoma, Enhanced by Lymph Node Status: An Integrative Approach
Source: Cancers (Basel). 2021 Jul 24;13(15):3722. doi: 10.3390/cancers13153722 (PMC8345215; doi:10.3390/cancers13153722)
Supplement: Supplementary file 1 [file cancers-13-03722-s001.zip › Supplementary files.pdf]

Supplemental Figure 1

a Oncomine Focus Assay

Hotspots in 35 genes			Copy number variants in 19 genes		Fusion drivers (23 genes)	
AKT1	FGFR2	MAP2K1	ALK	KIT	ABL1	FGFR2
ALK	FGFR3	MAP2K2	AR	KRAS	AKT3	FGFR3
AR	GNA11	MET	BRAF	MET	ALK	MET
BRAF	GNAQ	MTOR	CCND1	MYC	AXL	NTRK1
CDK4	HRAS	NRAS	CDK4	MYCN	BRAF	NTRK2
CTNNB1	IDH1	PDGFRA	CDK6	PDGFRA	EGFR	NTRK3
DDR2	IDH2	PIK3CA	EGFR	PIK3CA	ERBB2	PDGFRA
EGFR	JAK1	RAF1	ERBB2		ERG	PPARG
ERBB2	JAK2	RET	FGFR1		ETV1	RAF1
ERBB3	JAK3	ROS1	FGFR2		ETV4	RET
ERBB4	KIT	SMO	FGFR3		ETV5	ROS1
ESR1	KRAS		FGFR4		FGFR1	

b Ion AmpliSeq Colon and Lung Cancer Research Panel v2

Hotspots in 22 genes				
AKT1	EGFR	FGFR2	NOTCH1	STK11
ALK	ERBB2	FGFR3	NRAS	TP53
BRAF	ERBB4	KRAS	PIK3CA	
CTNNB1	FBXW7	MAP2K1	PTEN	
DDR2	FGFR1	MET	SMAD4	

Supplemental Figure 2

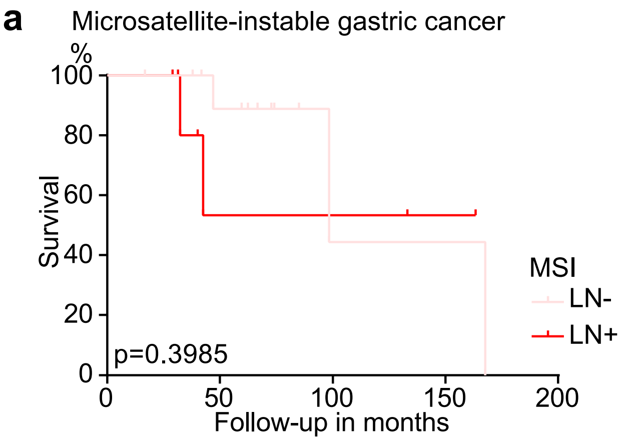

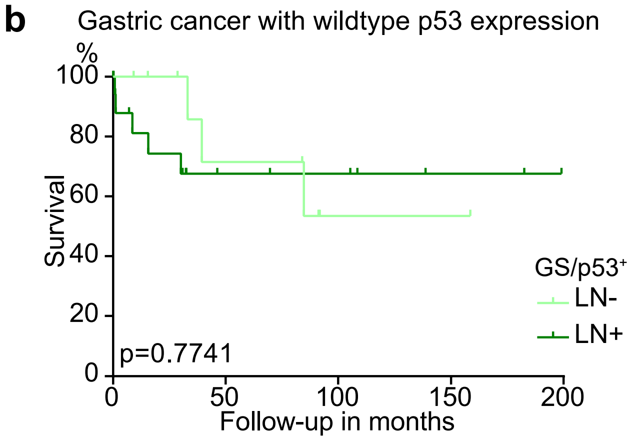

Supplemental Table 1: Histologic findings and clinical correlations in gastric adenocarcinoma

	EBER positive		MLH1 loss		E-cad loss		TP53		Lymph node metastases		*p value*
	n	%	n	%	n	%	n	%	n	%	
Total	2	1.7%	20	17.4%	10	8.7%	52	45.2%	72	62.6%	
**Gender**											
male	2	100.0%	10	50.0%	8	80.0%	34	65.4%	46	63.9%	0.5515
female	0	0.0%	10	50.0%	2	20.0%	18	34.6%	26	36.1%	
**Age (years)**											
median(min-max)	82 (61-91)		82 (61-91)		75 (44-81)		68 (44-89)		73 (44-90)		0.0003[a]
**Anatomic region**											
GEJ	0	0.0%	1	5.0%	0	0.0%	8	15.4%	6	8.3%	0.0532
Cardia	1	50.0%	2	10.0%	3	30.0%	20	38.5%	25	34.7%	
Corpus	1	50.0%	1	5.0%	0	0.0%	10	19.2%	11	15.3%	
Antrum	0	0.0%	16	80.0%	7	70.0%	14	26.9%	30	41.7%	
**Lauren classification**											
Intestinal	2	100.0%	17	85.0%	0	0.0%	40	76.9%	52	72.2%	0.6876
Diffuse	0	0.0%	1	5.0%	2	20.0%	5	9.6%	11	15.3%	
mixed	0	0.0%	2	10.0%	8	80.0%	7	13.5%	9	12.5%	
**WHO classification**											
Tubular	2	100.0%	17	85.0%	0	0.0%	39	75.0%	50	69.4%	0.7326
Papillary	0	0.0%	0	0.0%	0	0.0%	0	0.0%	1	1.4%	
Mucinous	0	0.0%	1	5.0%	1	10.0%	0	0.0%	2	2.8%	
Poorly cohesive	0	0.0%	0	0.0%	3	30.0%	7	13.5%	12	16.7%	
Mixed	0	0.0%	2	10.0%	6	60.0%	6	11.5%	7	9.7%	
**Pathologic T**											
1	0	0.0%	2	10.0%	0	0.0%	9	17.3%	5	6.9%	0.7458
2	1	50.0%	4	20.0%	1	10.0%	12	23.1%	12	16.7%	
3	0	0.0%	8	40.0%	4	40.0%	19	36.5%	26	36.1%	
4	1	50.0%	6	30.0%	5	50.0%	12	23.1%	29	40.3%	
**Pathologic N**											
0	1	50.0%	13	65.0%	3	30.0%	15	28.8%	0	0.0%	<0.0001
1+	1	50.0%	7	35.0%	7	70.0%	37	71.2%	72	100.0%	
**Pathologic N according to AJCC**											
0	1	50.0%	13	65.0%	3	30.0%	15	28.8%	0	0.0%	<0.0001
1	0	0.0%	2	10.0%	0	0.0%	12	23.1%	24	33.3%	
2	0	0.0%	3	15.0%	3	30.0%	9	17.3%	18	25.0%	
3	1	50.0%	2	10.0%	4	40.0%	16	30.8%	30	41.7%	
**Pathologic M**											
0	1	50.0%	20	100.0%	10	100.0%	48	92.3%	61	84.7%	0.1012
1	1	50.0%	0	0.0%	0	0.0%	4	7.7%	11	15.3%	
**AJCC stage**											
1	1	50%	5	25%	0	0%	14	27%	5	7%	0.0138
2	0	0%	9	45%	2	20%	12	23%	16	22%	
3	0	0%	6	30%	8	80%	22	42%	40	56%	
4	1	50%	0	0%	0	0%	4	8%	11	15%	

[a]one-way analysis of variance (ANOVA), for all other comparisons Chi-square test was used.
Numbers add up to more than 100% as individual patients are positive for more than one pathological or histological marker
